# Vitamin D and Cathelicidin (LL-37) Status in Patients with Type 2 Diabetes and *Staphylococcus aureus* Nasal Carriage

**DOI:** 10.1900/RDS.2021.17.30

**Published:** 2021-08-01

**Authors:** Marina N. Plataki, Rodanthi Vamvoukaki, George Samonis, Charalampos Bikis, Maria Gorgomiti, John A. Papadakis, Sofia Maraki, Diamantis P. Kofteridis

**Affiliations:** 1 Laboratory of Internal Medicine, Host Defense Unit, Faculty of Medicine, University of Crete, Heraklion, Crete, Greece,; 2 Department of Internal Medicine and Infectious Diseases, University Hospital of Heraklion, Heraklion, Crete, Greece,; 3 Department of Clinical Microbiology, University Hospital of Heraklion, Heraklion, Crete, Greece.

**Keywords:** cathelicidin, *Staphylococcus aureus* nasal carriage, vitamin D, type 2 diabetes

## Abstract

**OBJECTIVE:**

Type 2 diabetes mellitus (T2D) is characterized by the dysregulation of innate immunity leading to higher rates of *Staphylococcus aureus* nasal carriage, an important risk factor for severe infections. 25-hydroxy vitamin D (25(OH) D) may contribute, via the production of the antimicrobial peptide cathelicidin (LL-37), to epithelial host defense against *S. aureus*. This study evaluated whether 25(OH)D and LL-37 levels determine *S. aureus* nasal carriage.

**METHODS:**

Two consecutive nasal swabs were obtained from 118 T2D patients to determine *S. aureus* nasal carriage status. Serum levels of 25(OH)D and LL-37 were measured using chemiluminescence immunoassay and enzyme-linked immunosorbent assay, respectively. Supplementation of vitamin D by a number of participants was taken into account and evaluated.

**RESULTS:**

Forty-two T2D patients (35.6%) were found to be colonized by *S. aureus*. Vitamin D deficiency was detected in sixty-nine patients (65.7%). Median value for LL-37 in T2D patients was 0.89 ng/ml (range 0.05-8.62 ng/ml). Circulating levels of LL-37 were higher in nasal carriers compared to non-carriers (1.25 ng/ml vs 0.72 ng/ml; p < 0.001). No difference was found in serum 25(OH) D levels between carriers and non-carriers. 25(OH)D and LL-37 serum levels correlated positively in non-carriers, while the relationship was inversed in the carrier group. Vitamin D supplementation was not associated with lower incidence of *S. aureus* nasal carriage (p = 0.706).

**CONCLUSIONS:**

T2D patients presented decreased serum levels of 25(OH)D and LL-37, indicating a potential impairment of innate immunity. Expression of LL-37 may be induced by *S. aureus* nasal carriage among people with diabetes. Vitamin D supplementation did not influence *S. aureus* nasal colonization in T2D patients.

## 1 Introduction

*Staphylococcus aureus* (*S. aureus*), a human pathogen of major clinical significance, causes both community- and hospital-acquired infections with high morbidity and mortality and is associated with severe costs. This organism commonly colonizes the human skin and mucosa with the vestibulum nasi being the most frequently colonized site. Approximately 10 to 40 percent of people in the community have nasal carriage of *S. aureus*. This carriage represents a major risk for overt clinical infections or spread to other individuals [1-2]. Several studies have shown that elimination of *S. aureus* carriage from the anterior nares reduces the incidence of *S. aureus* infections [3].

It has been suggested that mechanisms leading to *S. aureus* nasal colonization are multifactorial, with the host’s immune response being the most important one [1-2, 4]. Innate immunity seems to be a key determinant of *S. aureus* nasal carriage status. Higher carriage rates have been found in patients with diabetes mellitus and are mainly attributed to dysregulation of innate immunity [5-7]. Furthermore, higher prevalence of colonization with methicillin-resistant strains (MRSA) has been observed in type 2 diabetes mellitus (T2D) patients [8]. It is possible that innate immune response prevents *S. aureus* invasion of the mucosa resulting in colonization or, even worse, infection. Mediators of innate mucosal host defense have been found in nasal secretions and include lactoferrin, lysozyme, and antimicrobial peptides. *S. aureus* nasal carriers may present dysregulation of these innate immunity factors in their nasal secretions [9].

Vitamin D, apart from its skeletal effects, plays an important regulatory role in the immune system’s physiology, especially in macrophage activation and differentiation as well as in regulation of the production of antimicrobial peptides in epithelia [10-12]. Serum circulating 25-hydroxy vitamin D [25(OH)D], a precursor of the active metabolite, is considered the most accurate marker of vitamin D status [11]. Vitamin D deficiency (< 20 ng/ml) has been associated with increased susceptibility to bacterial infections, including those caused by MRSA [13].

Vitamin D transcriptionally regulates the production of the human cathelicidin (known as LL-37) gene, an endogenous cationic, alpha-helical antimicrobial peptide, which is induced in response to sterile injury or infection. LL-37 has a central role in cutaneous host defense against S. aureus [14-15]. Furthermore, LL-37 is stored in secondary granules of neutrophils and is secreted into the blood upon pathogen invasion. It has been found that LL-37 is internalized through an endocytic process and traffics into lysosomes to enhance bactericidal activity against *S. aureus* in human macrophages [16]. To exert its effect against *S. aureus*, LL-37 can act synergistically with another class of antimicrobial peptides, the defensins [17]. Also, LL-37 promotes cytokine release and chemotaxis by activating formyl peptide receptor-like 1 [15, 18]. Interestingly, MRSA strains exhibited reduced susceptibility to LL-37, probably because of the positive net cell-surface charge [19].

In this context, vitamin D, which is a direct inducer of antimicrobial innate immunity in humans, and its downstream antimicrobial molecule, LL-37, may have a role in colonization with S. aureus. To date, limited information is available about potential links between vitamin D, LL-37 and *S. aureus* nasal carriage in T2D patients. Therefore, the aim of the study was to evaluate whether different degrees of expression of 25(OH)D and LL-37 could be primary determinants for different *S. aureus* carriage status in T2D patients.

## 2 Materials and methods

### 2.1 Study population

The study population comprised 118 Greek individuals with T2D attending the Internal Medicine Outpatient Clinic of the University Hospital of Heraklion, Crete, Greece. T2D was defined according to established criteria [20]. Patients’ characteristics were evaluated and included age, sex, diabetes duration, treatment, complications, comorbidities, smoking status, lipid profile, glycosylated hemoglobin, and the use of vitamin D supplements. Exclusion criteria were the presence of infections or the use of antimicrobial or immunosuppressive medication at the time of sampling.

### 2.2 Staphylococcus aureus isolation and identification and antimicrobial susceptibility testing

Both anterior nares were sampled with a sterile swab which was immediately placed in Amies transport medium (BioMerieux, Marcy L’Etoile, France). All specimens were kept at 4°C before being inoculated onto mannitol salt agar and Columbia agar with 5% sheep blood. The culture plates were incubated at 36°C for 48 h. S. aureus was identified on the basis of colony morphology, Gram stain, catalase test, coagulase test, and the API 20 Staph system (BioMerieux) by standard microbiological protocols. S. aureus isolates were screened for methicillin resistance by the disk diffusion method according to the Clinical and Laboratory Standards Institute recommendations [21]. From the two nasal cultures, obtained within a one-month interval, individuals were classified as persistent carriers if both cultures were positive, non-carriers if both cultures were negative, and intermittent carriers in cases where one culture was positive, while the other was negative.

### 2.3 25(OH)D assay

Serum samples were collected in summer during the same period of nasal swab sampling and stored at -80 °C until analysis. Serum 25(OH)D levels were evaluated with the Access 25(OH) Vitamin D Total assay by the UniCel DxI 800 Immunoassay System (Beckman Coulter, Inc., Brea, CA, USA). This two-step competitive binding immunoenzymatic assay used a 25(OH) vitamin D analogue, alkaline phosphatase conjugate, which was added and competed for binding to the immobilized monoclonal anti-25(OH) vitamin D. The amount of analyte in the sample was determined from a stored multi-point calibration curve. Vitamin D deficiency was defined as a 25(OH)D level below 20 ng/ml, insufficiency as a 25(OH)D level of 21-29 ng/ml and sufficiency as a 25(OH)D level of 30-100 ng/ml, according to the Endocrine Society’s guidelines [22].

### 2.4 LL-37 assay

LL-37 was evaluated by enzyme-linked immunosorbent assay (HK321; Human LL-37 ELISA Kit, Hycult Biotechnology, Uden, Netherlands), as per the manufacturer’s instructions. All specimens were centrifuged (1500 g, 15 min), and the supernatants were frozen. In particular, three coated 96-well plates detecting human LL-37 were used. Standards and 2x diluted samples were added to appropriate wells and incubated for 1 h at room temperature. Serial dilutions of reconstituted human LL-37 served as standard. Following incubation with biotinylated tracer antibody to LL-37 for 1 h at room temperature, streptavidinperoxidase was added for 1 h. Between all steps, the ELISA plates were washed 3 times with wash/dilution buffer. After incubation with tetramethylbenzidine substrate solution for 30 min, the reaction was stopped with 2% oxalic acid before reading at a wavelength of 450 nm. Intra-assay and inter-assay variations were 5.3% (range, 3.9 to 6%) and 6.1% (range, 1.1 to 6.5%), respectively. All samples were assayed in duplicates.

### 2.5 Statistical analysis

Continuous data following a non-normal distribution are reported as median (minimum-maximum value). Counts and corresponding percentages were calculated for categorical variables. Data were analyzed with non-parametric repeated measure ANOVA or unpaired t-test, as appropriate. Spearman’s rho test was used for correlation between continuous variables. All statistical analyses were carried out using SPSS 18.0 software (SPSS Inc., Chicago, IL, USA). The conventional level of p-value of <0.05 was considered to be significant.

### 2.6 Ethics

The study protocol, complying with the 1964 Helsinki Declaration, was approved by the Ethics Committee of the University Hospital of Heraklion and a written informed consent was obtained from each participant.

## 3 Results

One hundred and eighteen individuals, (32 (27.1%) males), with a median age of 67 (44-83) years, and T2D duration from diagnosis ranging from 1 to 40 (median 8) years, were enrolled in the study.

In the nasal swab sampling, 42/118 individuals (35.6%) were found to be colonized by S. aureus. In the colonized group, 19 (16.1%) were defined as persistent and 23 (19.5%) as intermittent. Their demographic, clinical, and laboratory characteristics are presented in Table 1. In an analysis of nasal carriers by susceptibility pattern of S. aureus, 6 individuals (14.3%) were colonized with MRSA and 33 (78.6%) with methicillin-sensitive S. aureus (MSSA). Three carriers (7.1%) appeared to have mixed colonization by MRSA and MSSA.

**Table 1.** Clinical characteristics and laboratory findings among persistent or intermittent *S. aureus* nasal carriers and non-carriers

Characteristics	Persistent carriers(n = 19)	Intermittent carriers(n = 23)	Non-carriers(n = 76)	p-value
Age (yr)	68 (44-80)	67 (47-78)	67 (47-83)	0.260
Male	4 (21.1%)	4 (17.4%)	24 (31.6%)	0.330
Diabetes duration (yr.)	10 (3-35)	7.5 (1-33)	8 (1-40)	0.401
*Diabetes treatment*				
Non-insulin treated	15 (78.9%)	21 (91.3%)	60 (78.9%)	0.394
Insulin treated	4 (21.1%)	2 (8.7%)	16 (21.1%)	
*Complications of diabetes*				
Cardiovascular disease	6 (31.6%)	6 (26.1%)	15 (19.7%)	0.503
Peripheral vascular disease	4 (21.1%)	2 (8.7%)	12 (15.8%)	0.528
Nephropathy	2 (10.5%)	3 (13%)	9 (11.8%)	0.969
Retinopathy	2 (10.5%)	1 (4.4%)	8 (10.5%)	0.658
Neuropathy	2 (10.5%)	0 (0%)	6 (7.9%)	0.326
*Concomitant underlying diseases*				
Hypertension	13 (68.4%)	18 (78.3%)	62 (81.6%)	0.454
Current smoking	2 (10.5%)	2 (8.7%)	4 (5.3%)	0.660
Total cholesterol	169 (113-256)	183 (118-271)	182 (90-277)	0.499
*HDL	48 (35-80)	49 (27-78)	48 (15-84)	0.938
**LDL	86 (44-177)	101 (66-181)	106 (57-178)	0.574
Triglycerides	116 (55-275)	160 (56-283)	137 (41-440)	0.149
***HbA1c	6.9 (6-11)	6.9 (5-9)	7.1 (6-12)	0.379
Vitamin D supplementation	4 (21%)	3 (13%)	14 (18.4%)	0.706
25(OH)D	15.5(0.8-45)	16.5 (4.6-32.4)	14.5 (4.5-42.2)	0.661
LL-37	1.61 (0.68-3.94)	1.24 (0.72-8.62)	0.72 (0.05-2.3)	<0.001

**Legend:** Significance level p < 0.05. P-values were calculated by chi-square test or t-test. Numerical variables are shown as median (minimum-maximum). *Abbreviations:* HDL - high-density lipoprotein, LDL - low-density lipoprotein, HbA1c - glycated hemoglobin.

Sixty-nine T2D patients (65.7%) presented 25(OH) D deficiency, while insufficiency was observed in 24 patients (22.9%). Among carriers, 17 (40.5%) presented vitamin D deficiency, while 8 (19%) had insufficiency. No difference was found in serum 25(OH)D levels between carriers (persistent or intermittent) and non-carriers. However, MSSA nasal carriers showed higher levels of 25(OH)D compared to MRSA carriers (p = 0.029).

**Figure 1.**
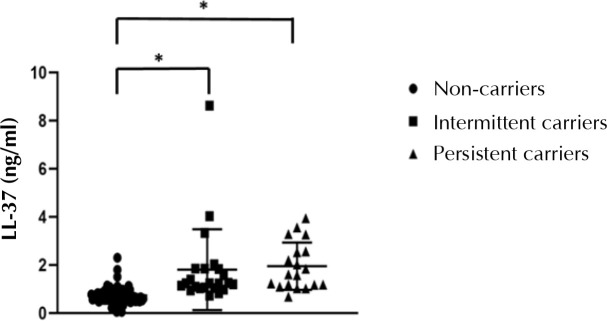
**Antimicrobial peptide cathelicidin (LL-37) in diabetic patients with persistent or intermittent *S. aureus* nasal carriage and non-carriage**. Serum LL-37 levels were significantly higher in the two carrier groups (persistent and intermittent) compared with non-carriers. There was no statistically significant difference between LL-37 levels in the two carrier groups. Statistical comparison was made using the Kruskal-Wallis test, * p < 0.001.

**Figure 2.**
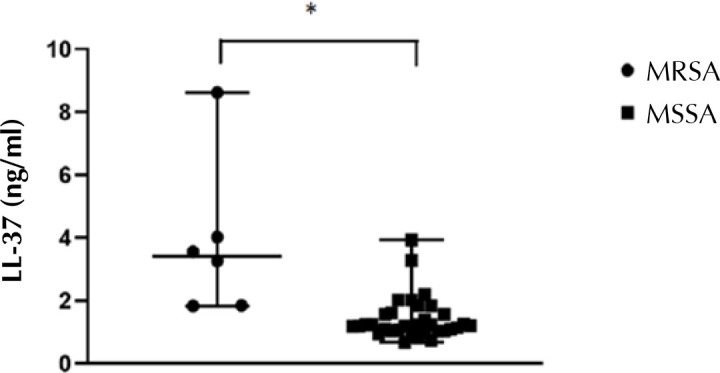
**Antimicrobial peptide cathelicidin (LL-37) and *S. aureus* antimicrobial susceptibility in nasal carriers**. Serum LL-37 levels were significantly higher in MRSA than MSSA nasal carriers. The Mann-Whitney test was used for statistical comparison, * p < 0.001.

Circulating levels of LL-37 in healthy subjects, obtained from previously published studies, are summarized in Table 2. In comparison with these findings, all 118 T2D patients from the present study had low serum LL-37 levels (median 0.89; range 0.05-8.62 ng/ml). However, nasal S. aureus carriers presented significantly higher serum LL-37 levels than non-carriers (p < 0.001) (Figure 1). Furthermore, MRSA carriers had higher levels of LL-37 than MMSA carriers (p < 0.001) (Figure 2).

**Table 2.** Circulating levels of LL-37 among healthy individuals from previously published studies

Blood levels (ng/ml)	Number of participants	ELISA kit	Reference
4.72 (4.57-4.88)*	18	Hycult Biotech	Honda *et al*. [23]
2.71 ± 3.57**	25	MyBioSource	Majewsky *et al*. [24]
7.92 (0.91-11.2)***	100	Hycult Biotech	Mutairi *et al*. [25]
27.2 ± 4.9**	21	Hycult Biotech	Jeng *et al*. [26]
32.20 ± 10.14**	30	Hycult Biotech	Zhan *et al*. [27]

**Legend:** * Mean (lower confidence interval, upper confidence interval), ** mean (± standard deviation), *** mean (range).

Twenty-one (17.8%) out of 118 T2D patients were receiving vitamin D supplementation (daily intake of at least 800 IU for at least 6 months). The median value of vitamin D in these patients was found to be 19.5 ng/ml. Vitamin D supplementation was not associated with lower incidence of S. aureus nasal carriage, neither persistent nor intermittent (p = 0.706).

**Figure 3.**
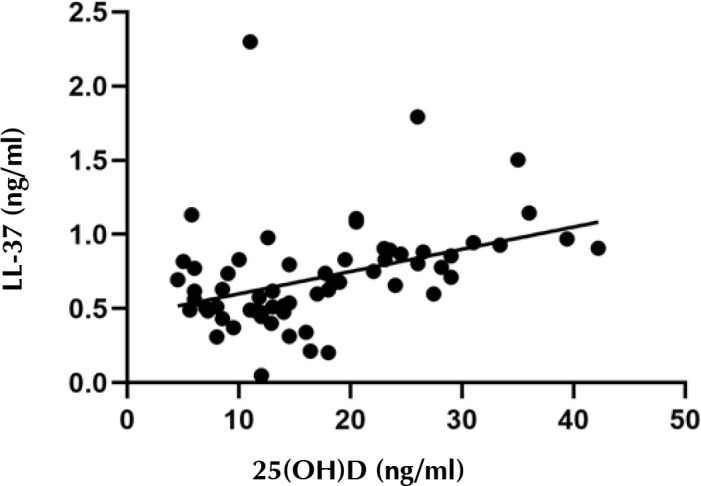
**Relationship between serum 25-hydroxyvitamin D and cathelicidin (LL-37) in non-carriers**. There was a strong positive correlation between vitamin D and LL-37 serum levels in non-carriers (coefficient = 0.48, p < 0.001). This relationship remained significant after adjustment for vitamin D supplementation (p = 0.002).

**Figure 4.**
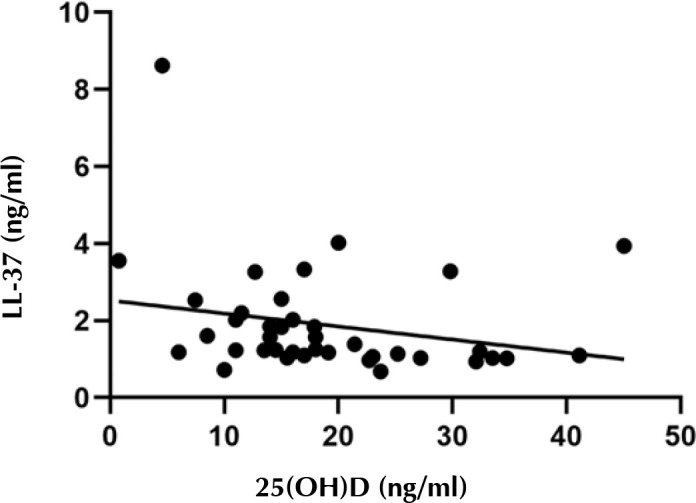
**Relationship between serum 25-hydroxyvitamin D and cathelicidin (LL-37) in *S. aureus* nasal carriers**. There was a moderate negative correlation between 25(OH)D and LL-37 in nasal carriers (coefficient = -0.392, p = 0.011).

Spearman’s analysis was used to investigate the correlation between 25(OH)D and LL-37 serum levels in T2D patients. A strong positive correlation was observed between vitamin D and LL-37 serum levels in non-carriers, as shown in Figure 3 (coefficient = 0.48, p < 0.001). This relationship was independent of vitamin D supplementation in a linear regression analysis (p = 0.002). In contrast, a moderate negative correlation was found between 25(OH)D and LL-37 among nasal carriers (coefficient = -0.392, p = 0.011) (Figure 4).

## 4 Discussion

The present study showed that T2D patients had decreased serum levels of 25(OH)D and LL-37, indicating a potential impairment of their innate immunity. Differential expression of LL-37 seems to be induced by different S. aureus nasal carriage status among T2D patients, while no effect of vitamin D administration on the rates of S. aureus nasal carriage was found.

The overall high rate of nasal carriage and the persistent colonization by S. aureus found in the present study are consistent with those reported in the literature [2, 28]. In total, 6 individuals (14.3%) were colonized with MRSA, representing a higher colonization rate in patients with T2D than in healthy adults [28]. Also, a meta-analysis conducted by Stacey *et al*. showed a higher prevalence of MRSA colonization among diabetic patients [8].

In recent years, knowledge of host factors influencing S. aureus nasal carriage has expanded significantly. Vitamin D exhibits various immunomodulatory effects by regulating innate immunity. In particular, vitamin D exerts anti-inflammatory actions via downregulating the expression of NFκB-dependent release of pro-inflammatory cytokines. Simultaneously, vitamin D positively regulates the production of LL-37 via the induction of vitamin D receptor [10]. Ninety-three T2D patients (88.6%) in the present study had either vitamin D deficiency or insufficiency. Of note, many human studies have shown an inverse association between vitamin D status and prevalence of T2D [29]. Therefore, it is plausible that metaflammation induced by T2D may result in negative regulation of the anti-inflammatory and immunomodulating actions of vitamin D or vice versa.

Nonetheless, the present study did not find a correlation between the levels of 25(OH)D and S. aureus nasal carriage status among people with diabetes. However, this might be related to the fact that the vast majority of patients had low vitamin D serum levels. This finding is supported by an observational study in diabetic patients, in which no connection between 25(OH)D levels in plasma and S. aureus carriage was observed [30]. Interestingly, when the level of 25(OH)D was compared in MRSA and MSSA carriers, decreased 25(OH)D levels in the MRSA group have been observed in the present study. A study using National Health and Nutrition Examination Survey data has shown that vitamin D deficiency increased the risk of MRSA, but not MSSA colonization, after adjustment for confounding factors [31]. If serum vitamin D level is, indeed, an acute phase reactant, we may consider that low serum vitamin D levels may be the result of MRSA carriage rather than a risk factor for it.

LL-37, produced mainly through a vitamin D-dependent mechanism, is a multifunctional modulator of cytokine secretion and adaptive immunity and is expressed by keratinocytes and immune cells [15]. In the present study, serum LL-37 concentrations were found to be low in T2D patients, suggesting a possible impairment of antimicrobial peptide production in the diabetic milieu. The finding may be insufficient to substantiate this conclusion. Therefore, further investigation, including non-diabetic subjects, is warranted. Higher concentrations of human cathelicidin LL-37 have been found previously in serum of healthy individuals [23-27, 32]. In accordance with the present findings, Gonzalez-Curiel et al. indicated that T2D patients have lower LL-37 gene expression [33]. This is in line with low or zero LL-37 expression levels in diabetic foot ulcer biopsies in comparison with those in healthy skin [34].

This is the first study that examined the association between LL-37 serum levels and S. aureus nasal carriage among T2D patients. In vitro LL-37 presents a remarkable killing efficacy against S. aureus [18]. In the present study, LL-37 serum levels were found to be higher among S. aureus nasal carriers compared to non-carriers in T2D patients. Higher LL-37 and betadefensin concentrations were also observed in nasal secretions among healthy S. aureus carriers compared to non-carriers [35-36]. Based on the findings described, elevated serum levels of LL-37 do not necessarily prime the host for an effective anti-staphylococcal response. It could be hypothesized that LL-37 expression is induced by S. aureus, but the concentration achieved, in the context of diabetes, may be too low to exert its antimicrobial activity. Higher serum levels of LL-37 do not necessarily correlate with increased epithelial levels. Instead, they may indicate an activated status of neutrophils in diabetic patients, who are prone to cell death by neutrophil extracellular traps (NETosis). Importantly, LL-37 is a major effector molecule that is released during NETosis. Collectively, higher serum LL-37 levels possibly reflect metaflammation rather than innate epithelial immunity.

Based on the antimicrobial susceptibility pattern, LL-37 levels were found to be higher in MRSA than MSSA carriers, although this finding is rather vague due to the small number of cases. There is evidence that MRSA strains possess an elevated resistance to endogenous antimicrobial peptides such as LL-37. Therefore, we may hypothesize that a much larger concentration of cathelicidin is needed to eliminate MRSA than that needed for MSSA [19, 37].

Another interesting finding of the present study is that, although circulating levels of 25(OH)D and LL-37 in non-carriers were positively correlated, their relationship was found to be inversed among carriers. These data suggest that LL-37 production during S. aureus colonization is regulated by vitamin D-dependent and -independent pathways, depending on the bacterial strain and host immune status. A study by Bhan et al. showed a positive correlation between 25(OH)D and LL-37 in healthy subjects only at 25(OH)D levels ≤ 32 ng/ml [38]. Another study by Adams et al. did not show a correlation between circulating levels of 25(OH)D and cathelicidin in an older population [39]. It is possible that additional factors confound the relationship between vitamin D and cathelicidin, e.g. renal insufficiency. In order to clarify better the interaction between vitamin D status and LL-37 responses within immune cells, localized or intracellular cathelicidin concentrations need to be assessed in detail.

Vitamin D supplements for restoration of defective innate immune functions have been a research subject in recent years. A number of studies have examined the role of vitamin D supplementation on S. aureus nasal carriage status. However, in the present study, no effect of daily vitamin D administration on the rates of S. aureus nasal carriage in T2D patients has been observed. Similarly, vitamin D supplementation did not reduce persistent S. aureus nasal carriage among healthy individuals in a study by Slow et al. [40]. Also, a randomized clinical trial showed that administration of vitamin D did not influence MRSA carriage [41].

The most important limitation of the present study is the relatively small sample size, especially the number of patients receiving vitamin D supplementation, a factor reducing its power. More studies in larger populations are needed to verify the present findings. Measurement of LL-37 in nasal epithelia of diabetic patients could be an interesting future continuation of the current work. Additionally, since multiple antimicrobial peptides act in synergy to clear S. aureus effectively, evaluation of their concentrations should also be performed in future studies for a better understanding of host innate response in T2D.

## 5 Conclusions

The present study has revealed low 25(OH)D and LL-37 serum levels in T2D patients. More studies are needed to determine the impact of low 25(OH)D and LL-37 on innate immunity of those patients.

S. aureus nasal carriers presented higher circulatory LL-37 levels than non-carriers. Moreover, no effect of daily vitamin D administration on the rates of S. aureus nasal carriage was found. Understanding the mechanisms by which vitamin D-cathelicidin regulates innate host defense systems in the context of diabetes is of prime importance for exploring candidates for host-directed therapeutics against staphylococcal infections.

## Abbreviations

ELISA enzyme-linked immunosorbent assay

25(OH)D 25-hydroxy vitamin D

ELISA enzyme-linked immunosorbent assay

LL-37 antimicrobial peptide cathelicidin

MRSA methicillin-resistant *Staphylococcus aureus*

MSSA methicillin-sensitive *Staphylococcus. aureus*

NETosis cell death by neutrophil extracellular traps

T2D type 2 diabetes mellitus
